# Complete mitochondrial genome of the Italian slow-worm *Anguis veronensis* Pollini, 1818, and its comparison with mitogenomes of other *Anguis* species

**DOI:** 10.1080/23802359.2017.1280705

**Published:** 2017-02-06

**Authors:** Tomasz Strzała, Renata Grochowalska, Bartłomiej Najbar, Angelica Crottini, Barbara Kosowska, Daniel Jablonski

**Affiliations:** a Department of Genetics, Wroclaw University of Environmental and Life Sciences, Wrocław, Poland;; b Department of Biochemistry and Bioinformatics, Faculty of Biological Sciences, University of Zielona Góra, Zielona Góra, Poland;; c Department of Botany and Ecology, Faculty of Biological Sciences, University of Zielona Góra, Zielona Góra, Poland;; d CIBIO Research Centre in Biodiversity and Genetic Resources, InBIO, Universidade do Porto, Porto, Portugal;; e Department of Zoology, Comenius University in Bratislava, Bratislava, Slovakia

**Keywords:** Mitogenome, anguidae, legless, lizards, Italian, peninsula, endemic, species

## Abstract

In this paper, we present complete mitochondrial genome of the Italian legless lizard species *Anguis veronensis* Pollini, 1818. The complete mtDNA consisted of 13 protein-coding genes, 22 tRNAs, and two rRNA genes which in total formed a DNA strand of 17,322 bp. *Anguis veronensis* mitogenome had the same gene order as two other compared *Anguis spp*., i.e. *A. cephallonica* and *A. fragilis*. The base composition of *A. veronensis* mitochondrial genome was A – 30.8%, T – 24.9%, C – 29.9%, G – 14.4%, with an A + T bias (55.7%). The newly described genome provides valuable data for future comparative mitogenomic analysis within *Anguis* genus.

The genus *Anguis* (slow worms) comprising a group of Palearctic legless lizards, has recently been the subject of numerous genetic studies (Gvoždík et al. 2010; Gvoždík et al. 2013; Mezzasalma et al. 2013). Four out of five currently described species occur in the Balkans and only two of them (*A. fragilis, A. colchica*) have a broad distributions (Jablonski et al. 2016). South European endemic *Anguis veronensis* Pollini, 1818, is mainly distributed, although it is not entirely restricted, to the Italian Peninsula. The species has been recently resurrected based on molecular (Gvoždík et al. 2013) and karyological (Mezzasalma et al. 2013) markers, despite its weak morphological differentiation from other slow-worm species (Gvoždík et al. 2013). The phylogenetic relationships within the genus *Anguis* are still largely unresolved (Gvoždík et al. 2013). Therefore, we sequenced the complete mitochondrial genome (mtDNA) of this ancient *Anguis* lineage to provide a valuable new tool to resolve phylogenetic relationships (Douglas et al. 2006; Douglas & Gower 2010; Nabholz et al. 2013). Data obtained in this study, along with already sequenced mitochondrial genomes of *Anguis* species (Albert et al. 2009; Strzała et al. 2016), will provide opportunity of comprehensive mitogenomic study of these legless lizards.

A tissue sample was collected from a road-killed individual in Bianzano (province of Bergamo; 45.774119°N, 9.921307°E) and is deposited in Zoological Collection of Angelica Crottini (ACZC3305) housed in CIBIO/InBIO, Research Centre in Biodiversity and Genetic Resources. ND2 sequence of this sample was already available and confirmed its taxon identity, which was also confirmed with Bayesian phylogenetic tree created with MrBayes 3.2.5 (Ronquist et al. 2012) (Figure 1). (Figure 1, KC881553). Total genomic DNA was isolated with Sherlock AX (A&A Biotechnology, Gdynia, Poland) according to the product manual. Mitochondrial genome was amplified with three overlapping fragments and sequenced using primer walking method by Wyzer Biosciences Inc. (Cambridge, MA). Mitochondrial genome was assembled with MITOS WebServer (Bernt et al. 2013) and checked manually.

**Figure 1.**
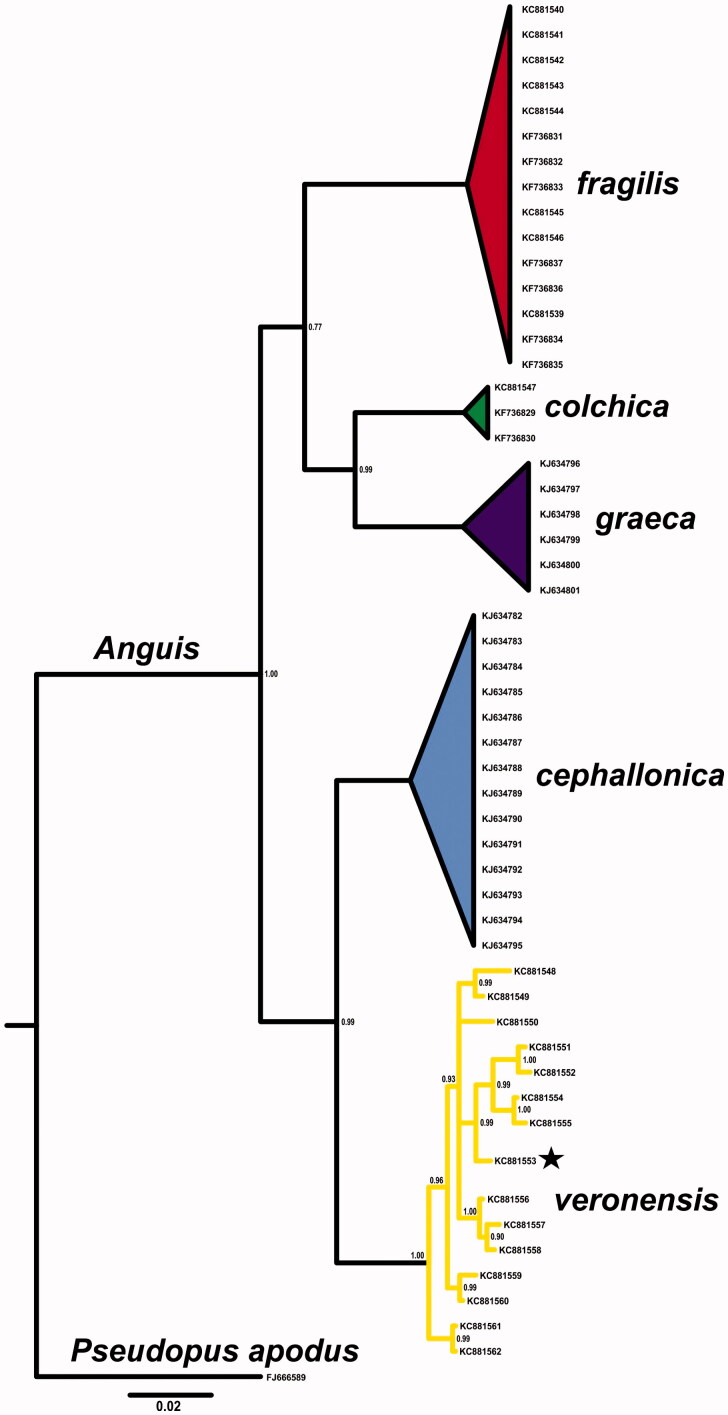
Bayesian phylogenetic tree of *Anguis* spp. representatives, created with 765 bp of *ND2* gene alignment. The tree was created using GTR + I +G model of substitution, as suggested by jModelTest 2.1.10 (Guindon & Gascuel 2003; Darriba et al. 2012). Tree was generated with 10,000,000 MCMC generations and 25% of burn-in. The individual used for mitogenome sequencing is marked with a star. A homologous sequence of *Pseudopus apodus* was used as an outgroup. Genbank accession numbers and Bayesian posterior probabilities of nodes are shown on the tree.

The complete mitochondrial genome of the *A. veronensis* (in total 17,322 bp; GenBank KX236332) has the same gene order and organization as mitogenomes of *A. fragilis* and *A. cephallonica*. Mitochondrial DNA of *A. veronensis* is shorter than mtDNA of the *A. fragilis* (17479 bp) but longer than the one of *A. cephallonica* (17208 bp). Differences in mtDNA length are the result of control region length polymorphism (1779 bp for *A. cephallonica*, 1891 bp for *A. veronensis*, and 2033 for *A. fragilis*). Overall base composition of *A. veronensis* H-strand is: A – 30.8%, T – 24.9%, C – 29.9%, G – 14.4%. In all three mitogenomes start codons have the same nucleotide composition with the exception of *ND3* and *ND5* (e.g.: ATG in *A. veronensis* and *A. cephallonica*; GTG in *A. fragilis*). As in *A. cephallonica,* six protein-coding genes truncated stop codons were identified in *A. veronensis* mitogenome, whereas only five truncated stop codons are present in *A. fragilis*. *Anguis veronensis* mitogenome has the highest A + T bias (55.7%) from all already known slow-worms mitochondrial genomes (54.7% for *A. cephallonica* and 55.2% for *A. fragilis*).
